# Effects of Poly(ethylene oxide) on the Foam Properties of Anionic Surfactants: Experiment and Molecular Dynamics Simulation

**DOI:** 10.3390/polym17172361

**Published:** 2025-08-30

**Authors:** Chaohang Xu, Ran Bi, Sijing Wang, Xiaojun Tang, Xiaolong Zhu, Guochun Li

**Affiliations:** 1 School of Safety Science and Emergency Management, Wuhan University of Technology, Wuhan 430070, China; xchwhut@whut.edu.cn (C.X.); br@whut.edu.cn (R.B.); mroirr@163.com (S.W.); 17723691699@163.com (X.T.); 2 School of Safety Engineering, China University of Mining and Technology, Xuzhou 221116, China; cqrczxl@163.com; 3 State Grid Shandong Electric Power Research Institute, Jinan 250000, China; 4 Shandong Smart Grid Technology Innovation Center, Jinan 250002, China

**Keywords:** poly(ethylene oxide), anionic surfactant, foaming ability, foam drainage half-life time, molecular dynamics simulation

## Abstract

Water-soluble polymers are often used as additives to adjust the foam properties of surfactant. In this study, the effects of water-soluble polymer poly(ethylene oxide) (PEO) on foam properties of two anionic surfactants, i.e., ammonium lauryl ether sulfate (ALES) and sodium dodecyl sulfate (SDS), were investigated by experimental and molecular dynamics simulation methods. Experimental results show that the addition of PEO can reduce the foaming ability of the two surfactants, but the inhibitory effect of PEO on the foaming ability is weakened at high surfactant concentration. Compared with ALES, PEO has a more significant inhibitory effect on the foaming ability of SDS. With the increase in PEO concentration, the half-life time of foam drainage in surfactant/water-soluble polymer composite systems gradually increases. The synergistic effect between PEO and ALES is stronger than that between PEO and SDS, resulting in a longer half-life time of foam drainage in ALES/PEO composite system. Molecular dynamics simulation results indicate that the addition of PEO can decline the air–water interface thickness of bubble films and the tail tilt angle of surfactant molecules at the air–water interface. The reduction in tail tilt angle means that the surfactant molecules are more vertical to the air–water interface and the hydrophobic interaction between adjacent tail chains of surfactants is weakened, which is unfavorable to the formation of bubble films, thus decreasing the foaming ability of surfactants. Because the ALES/PEO system has larger air–water interface thickness and surfactant tail tilt angle than the SDS/PEO system, the inhibitory effect of PEO on the foaming ability of ALES is weaker than that of SDS. Adding PEO can lower the peak position of the first hydration layer of surfactant head groups, increase the number of hydrogen bonds, and reduce the diffusion coefficient of water molecules, so that the surfactant/water-soluble polymer system has longer half-life time of foam drainage than the pure surfactant system. Due to the synergistic effect between ALES and PEO, the ALES/PEO system has a higher peak value of the first hydration layer of surfactant head groups, more hydrogen bonds, and lower diffusion coefficient of water molecules than the SDS/PEO system. Therefore, the half-life time of foam drainage in the ALES/PEO system is longer than that in the SDS/PEO system.

## 1. Introduction

Aqueous foam, as a gas–liquid two-phase medium, is widely used in firefighting [1], dust suppression [2], oil recovery [3], mineral flotation [4], cosmetics [5], the food industry [6], and so on. Due to the high surface tension, bubbles will burst immediately after formation, and pure water cannot generate stable foam. Surfactant with hydrophobic tail chains and hydrophilic head groups have to be added to water, thereby reducing surface tension to create foam. However, the performance of aqueous foam produced by pure surfactants often fails to meet the requirements of practical applications. For instance, fire-extinguishing foam requires high foam stability, while the foam generated by pure surfactants usually has poor stability [7]. Therefore, additives are often added to surfactant solutions to regulate the foaming ability and foam stability.

Among various types of additives, water-soluble polymers are effective substances for modifying the foam performance of surfactants [8]. Matouq et al. [9] investigated the effects of xanthan gum (XG) and polyvinyl pyrrolidone (PVP) on the foam properties of alkyl polyglycoside (APG) surfactant. The results showed that the type, concentration, and molecular weight of the water-soluble polymer significantly affected the foam stability. Momin et al. [10] studied the effects of nonionic water-soluble polymers, including polyethylene glycol (PEG), methyl cellulose, hydroxyl propyl cellulose, and polyvinyl pyrrolidone (PVP), on the foam performance of alpha olefin sulphonate (AOS) surfactant. It was found that at low AOS concentrations, an association between AOS and water-soluble polymers formed at the air–water interface, thereby reducing surface tension and enhancing the foaming ability, emulsifying property and solution viscosity. Wang et al. [11] tested the foam drainage rate of polyoxyethylene octylphenol ether-10 (OP-10) surfactant solutions with polyvinyl alcohol (PVA), polyacrylamide (PAM), and hydroxyethyl cellulose (HEC) water-soluble polymers. It was indicated that all three polymers could decline the foam drainage rate and improve the foam stability, and PAM performed the best. Li et al. [12] reported that low concentrations of carboxymethyl cellulose (NaCMC) improved the foaming ability of CTAB/501W surfactant solutions due to their interaction and increased viscosity, but high concentrations of NaCMC would inhibit the foaming ability. Additionally, NaCMC delayed the bubble coarsening and liquid drainage process, thereby improving foam stability. Figueredo et al. [13] evaluated the effect of poly(ethylene oxide) (PEO) on the stability of commercial aqueous film forming foam (AFFF) and found that PEO could prolong the existence time of foam, which may be caused by the polar interaction between the PEO ether group and surfactant head group. Li et al. [14] compared the effects of sodium alginate (SA) and polyvinyl alcohol (PVA) on the foam properties of dodecyl dimethyl betaine (BS-12) surfactant. The addition of PVA decreased the surfactant foaming ability and only improved the foam stability at high BS-12 concentrations, while SA had a synergistic effect with BS-12 and could enhance the foaming ability and foam stability at specific concentrations of BS-12. Mixtures of different surfactants with water-soluble polymers will result in different foam properties. Therefore, the influence of water-soluble polymers on the foam performance of surfactants is uncertain. Selecting the appropriate surfactants and water-soluble polymers to improve the foam properties remains a challenge.

The interaction between surfactants and water-soluble polymers at the gas–liquid interface is very complex, and it is precisely this interaction that leads to complex foam properties. Experimental methods cannot clarify the adsorption state and interaction between surfactants and water-soluble polymers at the gas–liquid interface from the microscopic level, but molecular dynamics (MD) simulation can. Amankeldiyeva et al. [15] investigated the bubble stability of water-soluble polymer XG and anionic surfactant SDS mixtures by MD simulation. At high concentrations, XG significantly inhibited the mobility of molecules such as surfactants, which helped to form a viscous interfacial liquid film, reduced the drainage rate, and enhanced the stability of bubbles. Wu et al. [16] employed MD simulation to study the mechanism by which PAM and partially hydrolyzed polyacrylamide (HPAM) affect the foam stability of SDS surfactant. The simulation results revealed that PAM forms hydrogen bonds with SDS head groups, thereby increasing the thickness of the hydration layer and slowing down the drainage rate. When the hydrolysis degree of HPAM reached 20%, the number of water molecules surrounding the surfactant head groups peaked, resulting in optimal foam stability. Xu et al. [17] conducted MD simulation on the bubble film formed by anionic surfactant sodium dodecyl ether sulfate (SDES) and water-soluble polymer PVA. They found that PVA enhanced the hydration of surfactant head groups and facilitated the formation of a three-dimensional hydrogen bond network among the surfactant, polymer, and water molecules, thus significantly improving the stability of the bubble film. Zhou et al. [18] discovered that the coexistence of surfactant APG, water-soluble polymer XG, and nanoparticles produced a strong hydrogen bond network in the liquid film, reducing the mobility of water molecules and increasing the foam stability. Therefore, it is extremely necessary to elucidate the interaction between surfactants and water-soluble polymers through MD simulation method, which can help reveal the underlying influence mechanism of water-soluble polymers on the foam properties of surfactants from the microscopic level.

In this work, poly(ethylene oxide) (PEO) was selected as the water-soluble polymer, because of its excellent environmental friendliness and few studies on the foam performance of PEO and surfactant mixtures. Anionic surfactants usually have good foaming performance. SDS is the most commonly used anionic surfactant in industrial foaming agents. Ammonium lauryl ether sulfate (ALES) has good environmental friendliness and low irritation and is a type of foaming agent with broad application prospects. Firstly, the influence of PEO on the foaming ability and foam drainage half-life time of the two surfactants was investigated by experimental tests. Then, through MD simulation, a bubble film model was constructed to analyze the air–water interface thickness, surfactant adsorption state, the radial distribution function (RDF) of water molecules around the surfactant head groups, hydrogen bond number, and mobility of water molecules for surfactant/water-soluble polymer composite systems. Furthermore, the differences in foam properties between ALES/PEO and SDS/PEO systems were explained from a microscopic perspective. This research helps to reveal the influence mechanism of water-soluble polymer PEO on the foam performance of anionic surfactants and provides theoretical guidance for screening surfactants and water-soluble polymers with synergistic effect.

## 2. Materials and Methods

### 2.1. Experimental Details

#### 2.1.1. Materials

**Figure 1:**
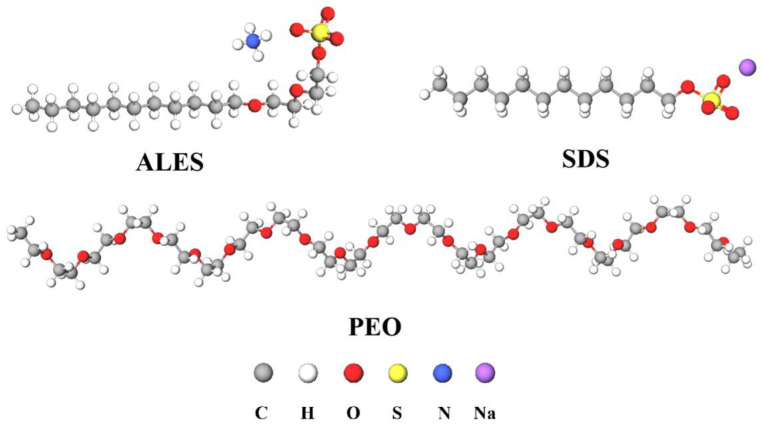
Molecular structures of ALES, SDS, and PEO.

Ammonium lauryl ether sulfate (ALES, purity ≥ 99%) was purchased from Shandong Usolf Chemical Technology Co. Ltd. Sodium dodecyl sulfate (SDS, purity ≥ 92.5%) was provided by Shanghai Macklin Biochemical Technology Co., Ltd. (Shanghai, China). Poly(ethylene oxide) (PEO, purity ≥ 98%) with a molecular weight of 60 × 10^4^ g mol^−1^, also known as epoxy [19], was purchased from Guangzhou Bohui Biotechnology Co., Ltd. The molecular structures of the three substances are shown in Figure 1. Distilled water was used in all experiments. All the solutions were stirred at 25 °C using a magnetic stirrer until the solutes were fully dissolved.

#### 2.1.2. Foaming Ability Measurement

**Figure 2:**
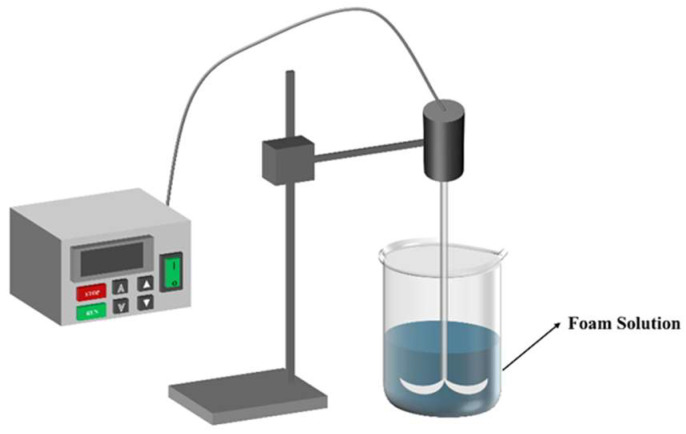
Schematic diagram of the foaming device.

Using the Waring–Blender method [20], a JJ-1B constant-speed electric stirrer was employed to generate foam. The schematic diagram of the foaming device is shown in Figure 2. The rotation speed was set to 1000 r/min and the stirring time was set to 1 min, so that all the 100 mL of the tested solution in the beaker participated in foam production. After stopping stirring, the foam in the beaker was immediately transferred to the measuring cylinder. The recorded foam volume V was used to characterize the foaming ability.

#### 2.1.3. Foam Drainage Half-Life Time Measurement

Start timing immediately when the stirring process was stopped. Then the liquid volume drained from the aqueous foam was recorded every second. When the drained liquid volume reached 50 mL, stop the timer and record the time as the half-life time of foam drainage.

### 2.2. Molecular Dynamics Simulation Details

#### 2.2.1. Simulation Model

**Figure 3:**
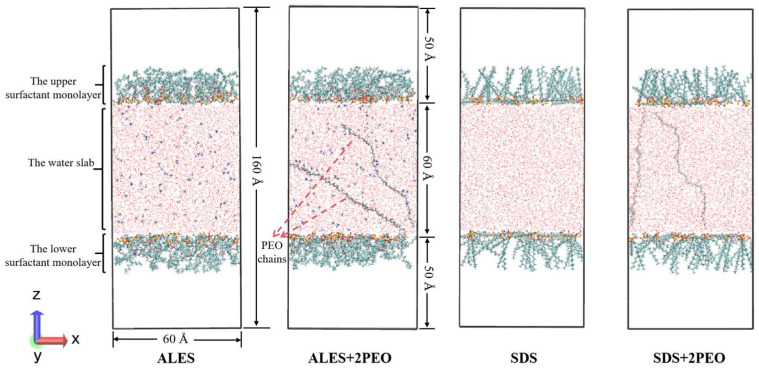
Initial configurations at the beginning of the simulations.

A sandwich model consisting of two surfactant layers and one water layer based on the real structure of bubble liquid films was constructed by Packmol program [21,22]. The length of the model in both x and y directions is 60 Å, and each of the upper and lower surfactant layers contained 60 surfactant molecules. The water layer was composed of 7000 water molecules and the cations of surfactant molecules. In the surfactant/water-soluble polymer composite systems, two PEO chains with a degree of polymerization of 20 were randomly added to the water layer. In order to eliminate the influence of periodic boundary conditions, two vacuum layers with 30 Å thickness were added below and above the surfactant layers, respectively. The surfactant molecules were arranged directionally and perpendicular to the xy plane. The hydrophilic head groups pointed towards the water layer and the hydrophobic tail chains faced towards the vacuum layer. Initial configurations at the beginning of the simulations are shown in Figure 3.

#### 2.2.2. Simulation Details

Gromacs software [23] version 2023.1 and OPLA-AA force field [24] were used to perform the simulations. Before each simulation, the system energy was minimized using the steepest descent algorithm with a tolerance of 1000 kJ·mol^−1^·nm^−1^. Then, the simulations were carried out in the NVT ensemble. The leapfrog algorithm was used to integrate the motion equations [25]. The time step was set to 2 fs and the total duration was 10 ns. Bond lengths were constrained using the LINCS algorithm [26]. The cutoff radius for short-range non-bonded interactions was set to 1.2 nm. The Ewald method was used to calculate the long-range electrostatic interactions with a cutoff radius of 1.2 nm [27]. The simple point charge (SPC) model was adopted for water molecules [28]. The temperature was set to 298 K using the Nosé–Hoover method with a temperature constant of 0.1 ps [29]. Periodic boundary conditions were employed in all directions. The trajectory was saved every 10 ps and visualized through VMD 1.9.3 software [30].

## 3. Results and Discussion

### 3.1. Experimental Results and Discussion

#### 3.1.1. Foaming Ability

**Figure 4:**
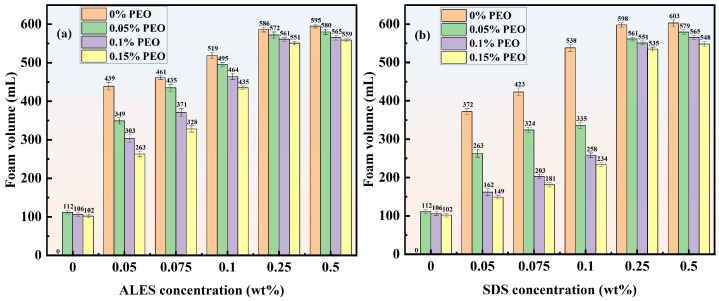
Foam volume of ALES/PEO and SDS/PEO composite solutions.

Figure 4 shows the foam volume of ALES/PEO and SDS/PEO composite solutions at different concentrations. It can be seen that the foam volume produced by pure PEO is approximately 100 mL, indicating that PEO has certain surface activity, but its foam volume is significantly lower than that of pure surfactants. As the surfactant concentration grows, the foam volume of surfactant/water-soluble polymer composite solutions exhibits an increasing trend. However, the addition of PEO reduces the foam volume of the two surfactants. At low surfactant concentrations (less than 0.1 wt%), the presence of PEO leads to a significant decline in foam volume. When the surfactant concentration is greater than 0.25 wt%, the inhibitory effect of PEO on the surfactant foaming ability decreases with the increase in surfactant concentration. The foam volume of surfactant/water-soluble polymer composite solutions gradually approaches that of pure surfactant solutions. The reason why PEO causes a decrease in the foaming ability is that the water-soluble polymer PEO has certain surface activity, which may form competitive adsorption with surfactant molecules at the air–water interface, thus hindering the generation of foam. However, when the surfactant concentration exceeds 0.25 wt%, the high concentration enables surfactant molecules to take the dominant role in the competitive adsorption. In this case, the foam volume is mainly determined by the surfactant concentration. The influence of PEO on the foaming ability becomes weak.

By comparing the foam volume of ALES/PEO and SDS/PEO systems, it can be observed that PEO inhibits the foaming ability of SDS more obviously than that of ALES. For example, when the surfactant concentration is 0.1 wt%, the foam volume of SDS solution without PEO is 538 mL. After adding 0.15 wt% PEO, the foam volume decreases to 234 mL, representing a reduction of 57%. For the 0.1 wt% ALES solution, the foam volume declines by only 16% after adding 0.15 wt% PEO. The weak inhibitory effect of PEO on ALES foaming ability may be related to the molecular structure of ALES. The ethylene oxide (EO) groups in ALES molecules may exhibit affinity interactions with PEO chains, thereby diminishing the inhibitory effect of PEO on the foaming ability. In contrast, the SDS molecule chain lacks hydrophilic EO groups. Consequently, at the same polymer concentration, the foaming performance of ALES solutions is relatively stable, while the foam volume of SDS solutions is significantly reduced.

#### 3.1.2. Foam Drainage Half-Life Time

**Figure 5:**
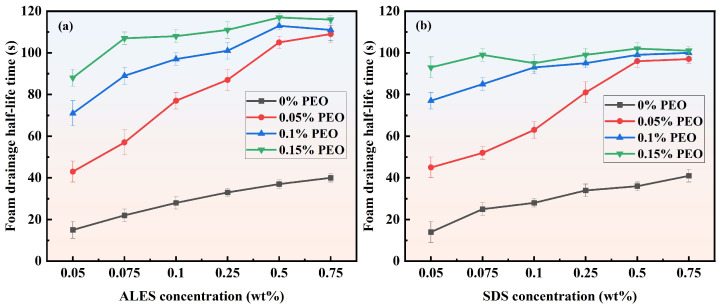
Foam drainage half-life time of ALES/PEO and SDS/PEO composite solutions.

Drainage is a crucial stage in the decay process of foam. During this stage, a large amount of water drains from the foam, causing the bubble films to become thinner. Then the foam stability decreases, the bubble size grows, and it enters the Oswald ripening stage. The half-life time of foam drainage in ALES/PEO and SDS/PEO systems is shown in Figure 5. As the surfactant concentration increases, the half-life time of foam drainage in the composite systems exhibits a trend of rapid growth at the beginning and then stabilizing eventually. With the increase in PEO concentration, the half-life time of foam drainage gradually increases, meaning that the foam stability is enhanced. It should be noted that for pure surfactant solutions, the foam drainage half-life time of ALES and SDS is almost the same. However, after adding the water-soluble polymer, the foam drainage half-life time of ALES/PEO composite systems is significantly higher than that of SDS/PEO composite systems. For instance, when the ALES concentration is 0.5 wt% and the PEO concentration is 0.15 wt%, the foam drainage half-life time of ALES/PEO composite system is the largest, which is 117 s, while the foam drainage half-life time of SDS/PEO composite system is only 100 s at the same concentrations, which is 17% shorter than that of the ALES/PEO system. This indicates that compared with SDS, PEO has a stronger synergistic effect with ALES, so the ALES/PEO composite system can delay the foam drainage process more effectively, resulting in higher stability of the ALES/PEO foam.

### 3.2. Simulation Results and Discussion

#### 3.2.1. Air–Water Interface Thickness

**Figure 6:**
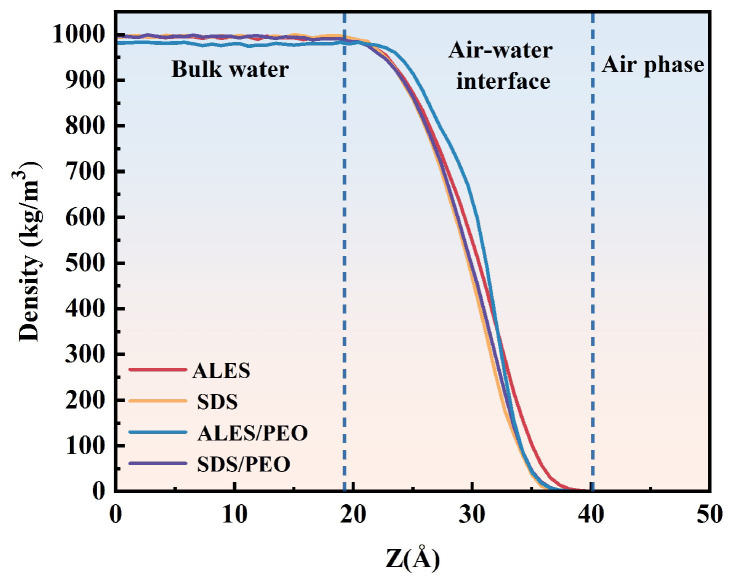
Density distribution of water molecules at the air–water interface.

The density distribution of water molecules at the air–water interface is shown in Figure 6. It can be seen that the content of water molecules gradually decreases from the bulk phase density to zero in the air phase. The air–water interface thickness is defined as the width from 10% to 90% of water density [31]. After calculation, the thicknesses of the air–water interface for the four systems are shown in Table 1. It can be observed that the air–water interface thicknesses of the surfactant/water-soluble polymer systems is lower than those of pure surfactant systems. After the addition of PEO, the air–water interface thickness of ALES decreases from 10.78 Å to 9.24 Å, while that of SDS declines from 9.24 Å to 8.47 Å. This indicates that PEO changes the adsorption state of surfactant molecules at the air–water interface. In addition, as a water-soluble polymer, the PEO chain contains multiple hydrophilic EO groups. These EO groups can interact with water molecules and attract some water molecules from the air–water interface to migrate around the polymer, thus reducing the thickness of the air–water interface. The thinning of the air–water interface thickness may be the reason for the decrease in foaming ability. Since the air–water interface thickness of ALES is higher than that of SDS—and even after the addition of PEO, the air–water interface thickness of ASLE/PEO system is still greater than that of SDS/PEO system—the inhibitory effect of PEO on the foaming ability of ALES is lower than that of SDS.

#### 3.2.2. Tilt Angle Distribution of Surfactant Tail Chains

**Figure 7:**
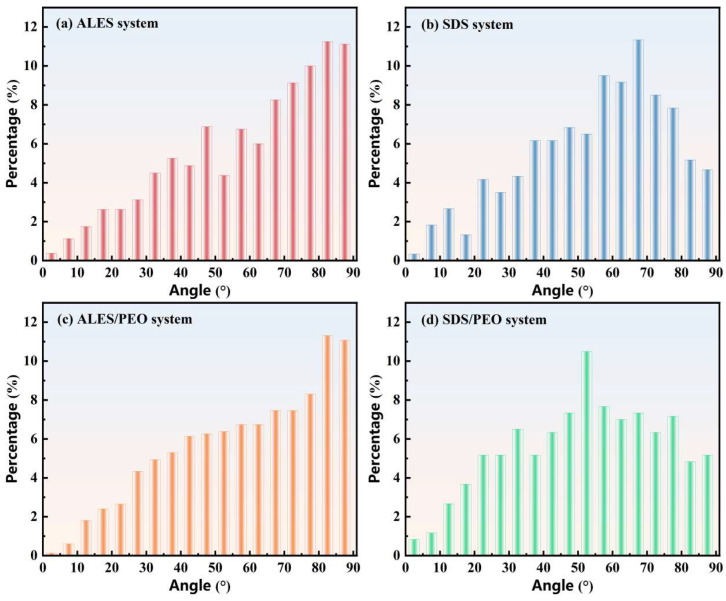
Tilt angle distribution of surfactant tail chains at the air–water interface.

In order to analyze the differences in the adsorption states of surfactant molecules at the air-water interface after the addition of PEO, the tilt angle distribution of surfactant tail chains in the pure surfactant and surfactant/water-soluble polymer systems were studied, as shown in Figure 7.

From Figure 7a,c, it can be seen that the tile angles of ALES tail chains decrease after the addition of PEO. The proportion of ALES molecules with tail chain tilt angles ranging from 70° to 90° declines from 41.5% to 38.1%. After adding PEO to the SDS solutions, the tilt angles of SDS tail chains are also reduced, as shown in Figure 7b,d. In the pure SDS system, the tail chain tilt angle of 67.5° accounts for the highest proportion of 11.33%, while in the SDS/PEO composite system, the tail chain tilt angle of 52.5° has the highest ratio of 10.5%. Moreover, after adding PEO, the average tilt angle of ALES tail chains decreases from 59.87° to 58.99°, and that of SDS tail chains drops from 55.03° to 51.79°, indicating that the addition of PEO can change the adsorption states of surfactant molecules. The tail chain tilt angles of the two surfactants both decrease after the addition of PEO, which means that the surfactant molecules are more vertical to the air–water interface, and the hydrophobic interaction between adjacent surfactant tail chains is weakened. It is unfavorable to the formation and stability of bubble films, thus reducing the foaming ability of surfactants.

As can be seen from Figure 7c,d, the tilt angles of surfactant tail chains in the ALES/PEO system is significantly larger than those in the SDS/PEO system. After calculation, the average tilt angle of surfactant tail chains in the ALES/PEO and SDS/PEO systems is 58.99° and 51.79°, respectively. This demonstrates that in the surfactant/water-soluble polymer system, ALES tail chains tend to be parallel to the air–water interface, while SDS tail chains are relatively vertical. The parallel arrangement of surfactant molecules helps to form an interlaced grid structure of tail chains, which enhances the interaction among surfactant molecules and delays the rupture of bubbles. Meanwhile, the interlaced grid structure of tail chains can effectively slow down the air permeation through the bubble films and improve the foam stability. However, the vertical arrangement of surfactant tail chains is not conducive to the formation of a dense molecular network structure, and the rupture resistance of the bubble films is weakened. Therefore, the foam stability of the ALES/PEO system is superior to that of the SDS/PEO system.

#### 3.2.3. Radial Distribution Function

**Figure 8:**
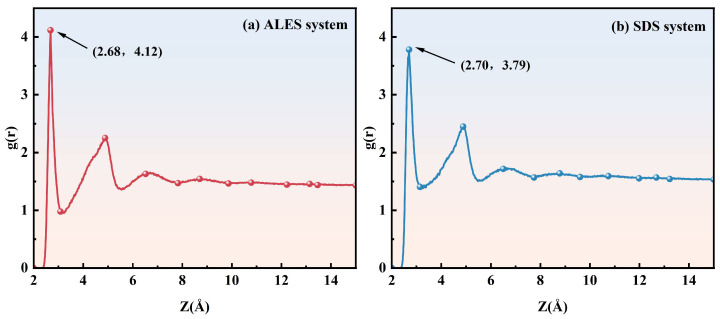

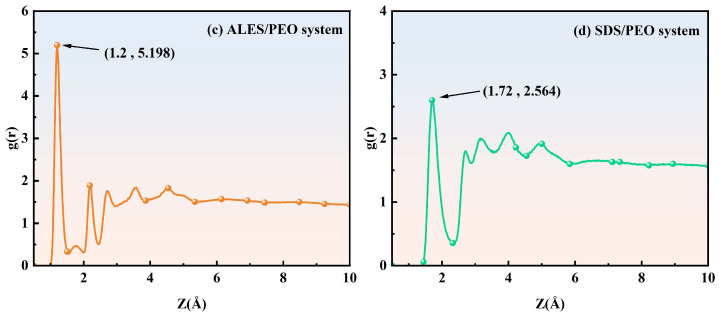
Radial distribution function curves of water molecules around the surfactant head groups.

Figure 8 shows the radial distribution function (RDF) curves of water molecules around the surfactant head groups. The position and peak value of the first hydration layer of the surfactant head groups in surfactant and surfactant/water-soluble polymer systems are given in Table 2. In the ALES/PEO composite system, the first hydration layer is located at 1.2 Å, which is lower than the position of 2.68 Å in the ALES system, indicating that the addition of PEO reduces the binding distance between water molecules and ALES head groups. For the SDS/PEO composite system, the first hydration layer of SDS head groups is situated at 1.72 Å, which is also lower than the value of 2.70 Å in the pure SDS system, yet still higher than the position of the first hydration layer in the ALES/PEO system. After adding PEO, the peak value of the first hydration layer of ALES head groups increases from 4.12 to 5.2, while that of SDS head groups declines from 3.79 to 2.56, showing that the addition of PEO can enhance the hydration strength of ALES groups, but will destroy the interaction between SDS head groups and water molecules. Therefore, the introduction of PEO can change the interaction between water molecules and surfactant hydrophilic head groups. Due to the smaller position and higher peak value of the first hydration layer, the hydration effect of surfactants in the ALES/PEO system is stronger than that in the SDS/PEO system, resulting in a higher degree of aggregation of water molecules around the surfactant head groups and less loss of water molecules. Therefore, the foam drainage half-life time of the ALES/PEO system is longer than that of the SDS/PEO system in the experiments.

**Figure 9:**
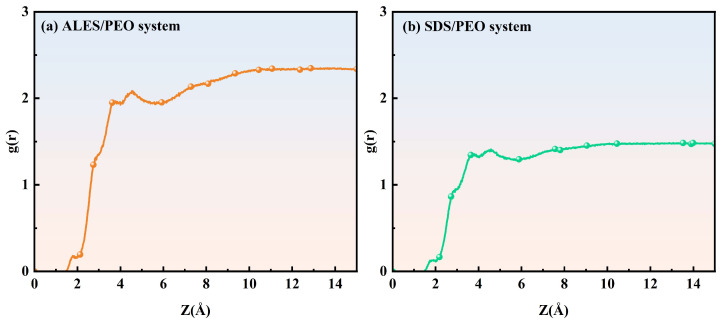
Radial distribution function curves of water molecules around the PEO chain.

The radial distribution function curves of water molecules around the PEO chain in ALES/PEO and SDS/PEO composite systems are shown in Figure 9. It can be seen that the RDF curves of the two composite systems exhibit the same trend, suggesting that the distribution characteristics of water molecules around the PEO chain are similar. However, the RDF value of water molecules around the PEO chain in the ALES/PEO system is higher than that in the SDS/PEO system at the same position. This demonstrates that in the ALES/PEO system, the interaction between water molecules and PEO is stronger, and water molecules are more likely to aggregate with the PEO chain, which is helpful to delay the drainage process of aqueous foam. Therefore, in the two surfactant/water-soluble polymer composite systems, ALES and PEO have a good synergistic effect, and the ALES/PEO system has stronger foam stability than the SDS/PEO system.

#### 3.2.4. Hydrogen Bond

**Figure 10:**
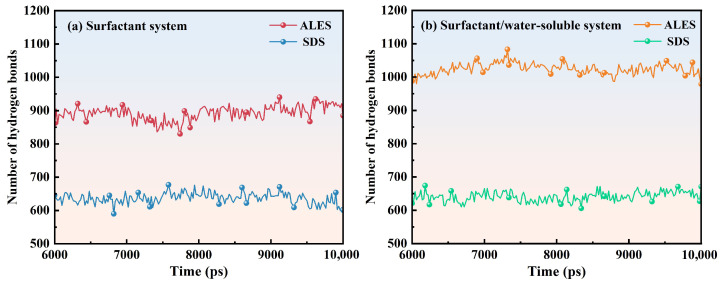
Hydrogen bond numbers formed between surfactants and water molecules.

**Figure 11:**
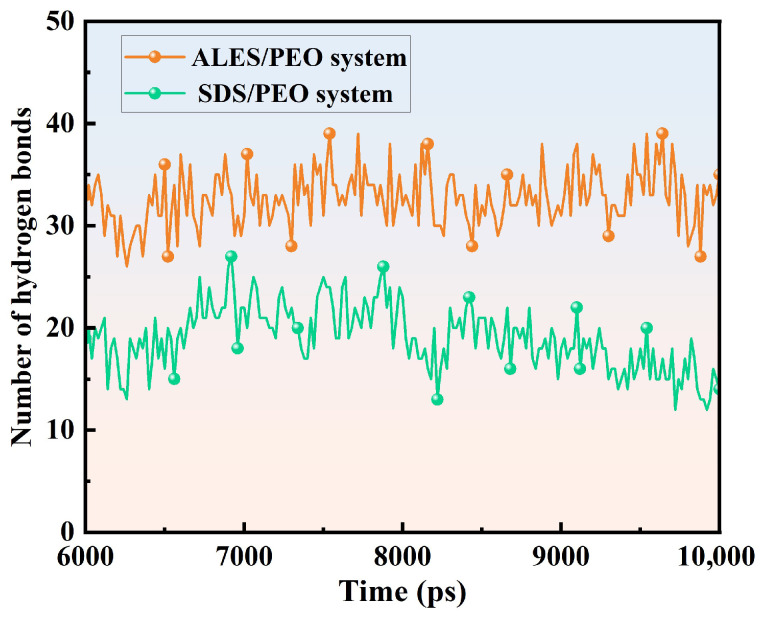
Hydrogen bond numbers formed between PEO and water molecules.

The number of hydrogen bonds (H-bonds) formed between surfactants and water molecules is presented in Figure 10 and Table 3. The number of H-bonds formed between the PEO chain and water molecules is also shown in Figure 11 and Table 3. After adding PEO, the number of H-bonds between ALES and water molecules significantly grows, with the number of H-bonds formed by per surfactant molecule increasing from 7.42 to 8.53, while the number of H-bonds between SDS and water molecules slightly rises, with the number of H-bonds formed by per SDS molecule increasing by only 0.08. This suggests that ALES and PEO have strong synergistic effect and significantly enhances the hydration of ALES surfactants. In the ALES/PEO composite system, the number of H-bonds between PEO and water molecules is 32.94, which is notably higher than the number of H-bonds (18.91) in the SDS/PEO system. This demonstrates that the synergistic effect between ALES and PEO also strengthens the interaction between PEO and water molecules.

#### 3.2.5. Mobility of Water Molecules

**Figure 12:**
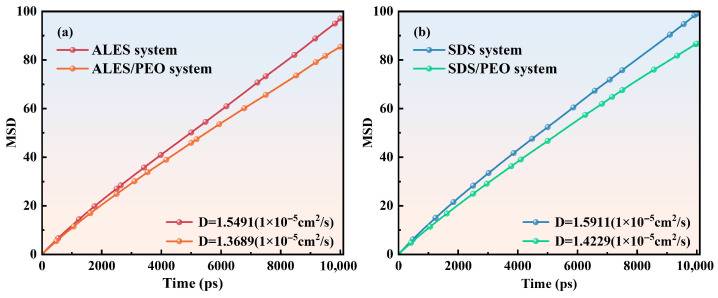
Mean square displacement of water molecules in different systems.

To investigate the influence of PEO on the mobility of water molecules in the surfactant/water-soluble polymer composite systems, the diffusion behavior of water molecules was analyzed using the mean square displacement (MSD) method, as shown in Figure 12. The diffusion coefficient (D) represents the mobility of water molecules in different systems. In the pure surfactant system, the diffusion coefficient of water molecules is large, and the degree of constraint is low. This leads to the easy drainage of water molecules from the liquid film, which is not conducive to the stability of foam. With the addition of PEO, the diffusion coefficient of water molecules in the surfactant/water-soluble polymer composite systems is significantly reduced. For ALES and SDS, the diffusion coefficients of water molecules decrease from 1.55 and 1.59 in pure surfactant systems to 1.37 and 1.42 in surfactant/water-soluble polymer systems, respectively. The addition of PEO decreases the diffusion rate of water molecules, which indicates that the resistance of water molecule movement is enhanced. This is because the presence of PEO increases the number of H-bonds in the surfactant/water-soluble polymer systems, thereby restricting the diffusion movement of water molecules. The weak diffusion ability slows down the foam drainage rate, enabling a long period of foam stability. Furthermore, the diffusion coefficient of water molecules in the ALES/PEO systems is lower than that in the SDS/PEO system. This is the reason why the foam drainage half-life time of the ALES/PEO system is higher than that of the SDS/PEO system.

## 4. Conclusions

Based on experiments and molecular dynamics simulations, the influence of water-soluble polymer PEO on the foam properties of two anionic surfactants (ALES & SDS) was explored. The simulation results are used to explain the changes in foam properties from a microscopic perspective. The main conclusions are as follows:

At low surfactant concentrations (less than 0.1 wt%), the addition of PEO leads to a significant decrease in foam volume. When the surfactant concentration is higher than 0.25 wt%, the foam volume of the surfactant/water-soluble polymer composite systems gradually approaches that of the pure surfactant system with the increase in surfactant concentration. Although the presence of PEO can reduce the foaming ability of both surfactants, the inhibitory effect of PEO on SDS is stronger than that on ALES. As the PEO concentration rises, the foam drainage half-life time of the two surfactants gradually increases, and the foam stability is enhanced. However, the half-life time of foam drainage in the ALES/PEO systems is longer than that in the SDS/PEO systems. This indicates that the synergistic effect between PEO and ALES is stronger than that between PEO and SDS, and the ALES/PEO system can delay the foam drainage process more effectively.

The simulation results show that the addition of PEO can reduce the air–water interface thickness of the bubble film and the tail tilt angles of surfactant molecules, resulting the decline of the foaming ability of surfactant solutions. The ALES/PEO composite system exhibits a thicker air–water interface layer compared to the SDS/PEO system. Moreover, the tail tilt angles of surfactant molecules in the ALES/PEO system are greater than those in the SDS/PEO system. This parallel arrangement of ALES molecules helps to form an interlaced grid structure of tail chains and enhances the interaction among surfactant molecules, thus slowing down the air permeation through bubble films and improving the foam stability. Compared with the SDS/PEO system, the ALES/PEO system has a smaller position and higher peak value of the first hydration layer of surfactant head groups. This stronger hydration effect results in a larger number of hydrogen bonds in the ALES/PEO system than that in the SDS/PEO system, suggesting that a strong synergistic effect is generated between ALES and PEO. Furthermore, compared with pure surfactant systems, the presence of PEO increases the number of hydrogen bonds in the surfactant/water-soluble polymer composite systems and decreases the diffusion coefficient of water molecules, thereby enhancing the resistance to the mobility of water molecules. The diffusion coefficient of water molecules in the ALES/PEO system is lower than that in the SDS/PEO system, which is also the reason why the foam drainage half-life time of the ALES/PEO system is longer than that in the SDS/PEO system.

## Figures and Tables

**Table 1:** Air–water interface thicknesses in the ALES, ALES/PEO, SDS, and SDS/PEO systems.

System	Position of the Air–Water Interface		Air–Water Interface Thickness (Å)
	Starting Point (Å)	Ending Point (Å)	
ALES	24.26	35.04	10.78
ALES/PEO	24.26	33.50	9.24
SDS	24.26	33.50	9.24
SDS/PEO	25.03	33.50	8.47

**Table 2:** The position and peak value of the first hydration layer of surfactant head groups.

System	Position of the First Hydration Layer (Å)	Peak Value of the First Hydration Layer
ALES	2.68	4.12
ALES/PEO	1.20	5.20
SDS	2.70	3.79
SDS/PEO	1.72	2.56

**Table 3:** Hydrogen bond numbers in the ALES, ALES/PEO, SDS, and SDS/PEO systems.

System	Number of Hydrogen Bonds	
	Per surfactant Molecule	Per PEO Chain
ALES	7.42	/
ALES/PEO	8.53	32.94
SDS	5.33	/
SDS/PEO	5.41	18.91

## Data Availability

The original contributions presented in this study are included in the article. Further inquiries can be directed to the corresponding author.
